# From conventional to disruptive: upturning the HIV testing status quo among men who have sex with men in Vietnam

**DOI:** 10.1002/jia2.25127

**Published:** 2018-07-22

**Authors:** Kimberly E Green, Bao N Vu, Huong TT Phan, Minh H Tran, Huu V Ngo, Son H Vo, Trang M Ngo, Anh H Doan, Tham T Tran, Trang NN Nguyen, An Bao, Lan TX Hang, Thanh M Le, Tung T Doan, Linh H Dang, Giang TT Ha

**Affiliations:** ^1^ PATH Viet Nam; ^2^ Ministry of Health/Vietnam Administration of HIV/AIDS Control Viet Nam; ^3^ Center for Creative Initiatives in Health and Population Viet Nam; ^4^ United States Agency for International Development Viet Nam; ^5^ Life Center Viet Nam; ^6^ G‐link Social Enterprise Viet Nam; ^7^ Lighthouse Social Enterprise Viet Nam

**Keywords:** MSM, HIV testing, community, HIV self‐testing, HIV lay provider testing, HIV cascade, social media, Vietnam

## Abstract

**Introduction:**

HIV prevalence among men who have sex with men (MSM) in Vietnam is increasing, while annual HIV testing uptake has remained consistently low, posing a significant challenge to reaching the 90‐90‐90 goals. Barriers to MSM seeking HIV testing include concerns regarding confidentiality and lack of convenient testing options. Two new HIV testing strategies—HIV lay provider and HIV self‐testing (HIVST)—were piloted alongside intensive social media outreach to increase access to and uptake of HIV testing among MSM not actively engaged in services.

**Methods:**

We measured the proportion of first‐time MSM HIV testers opting for HIV lay or self‐testing, and factors that were associated with first‐time testing, as part of a larger HIV lay and self‐testing study among key populations in Vietnam. We also assessed MSM satisfaction with HIV lay or self‐testing, and testing location and provider preferences. Finally, we calculated linkage to care cascade among MSM that were diagnosed and enrolled in anti‐retroviral therapy (ART) services.

**Results:**

Among MSM that sought HIV lay and self‐testing, 57.9% (n = 320) and 51.3% (n = 412) were first‐time testers respectively. In the final adjusted models, the odds of being a first‐time tester and opting for HIV lay testing were higher among MSM who were young, had lower levels of income and had never exchanged sex for money; for HIVST, the odds of being a first‐time HIV tester were higher among MSM that had attained lower levels of education. HIV lay and self‐testing resulted in higher detection of new HIV cases (6.8%) compared to conventional HIV testing among key populations (estimated at 1.6% in 2016), while MSM linked to testing through social media interventions presented with even higher HIV‐positivity (11%). Combined, 1655 HIV cases were diagnosed and more than 90% were registered for ART services.

**Conclusions:**

Our findings suggest that MSM‐delivered HIV testing and self‐testing, promoted through online or face‐to‐face interactions, offer important additions to MSM HIV testing services in Vietnam, and could significantly contribute to epidemic control by increasing HIV testing among harder‐to‐reach and higher‐risk MSM, effectively enrolling them in ART, and reducing onward transmission.

## 1 Introduction

Since 2002, the Vietnam Ministry of Health (MOH) has led a large‐scale HIV prevention, testing, and treatment programme primarily focused on reaching people who inject drugs (PWID), female sex workers (FSW), and, only more recently, men who have sex with men (MSM) 1. HIV prevalence among PWID and FSW has declined significantly over the past decade 2, 3. Among MSM, however, HIV prevalence has increased, from 4% in 2011 to 7.4% in 2016, with the highest HIV prevalence (13%) in Ho Chi Minh City (HCMC) 2.

Until recently, HIV testing was solely available in clinical settings, with a focus on client‐ and provider‐initiated facility‐based modalities. In addition, confirmation of HIV diagnosis was only allowed in MOH‐certified reference laboratories, which contributed to significant time delays in treatment enrolment 4.

Although the number of HIV testing sites has increased significantly, annual HIV testing uptake has remained low in MSM 5. Reported annual HIV testing among MSM was 30.2% in 2011, 28.2% in 2013 and 32% in 2015 6, 7. A national internet‐based survey among MSM found that 76.5% had never tested for HIV 8. The study revealed that common reasons for MSM not seeking an HIV test included feeling ashamed, fear of receiving a positive result and self‐perception of not being at risk of HIV. A representative cross‐sectional study in four high‐prevalence provinces reported that the primary features that MSM preferred in HIV testing were confidentiality and convenience 9. This same study measured acceptability of HIV self‐testing (HIVST) if it were available; 64% of MSM responded that they would opt for it, with privacy, confidentiality, speed and convenience being its primary advantages over conventional HIV testing 9. A qualitative study with MSM in HCMC also described a strong preference for HIV testing services offered in an MSM‐friendly environment 10. Meta‐analyses of HIV lay and self‐testing have found that, when compared to conventional HIV testing, these strategies can significantly increase uptake of HIV testing among new testers, boost frequency of testing, and better reach those that are at risk of HIV 11, 12, 13.

Another barrier to MSM HIV testing in Vietnam involves the limitations of face‐to‐face peer outreach. There are an estimated 330,000 MSM in Vietnam, of whom some 26% are considered reachable through traditional peer interventions 14. Studies measuring MSM online behaviour, social media consumption and service utilization suggest that a much larger population could be reached through online interactions 8, 15, 16. In a representative sample of MSM across four cities, 88.9% of MSM reported owning a smart phone, 97.6% had a social media account and 80% used Facebook regularly 9. Likewise, from an online descriptive cross‐sectional study among MSM in Hanoi, 66.2% of respondents stated they already used the Internet to seek HIV prevention information, and 73.4% said they sought sex partners online 16. Studies in Ghana, Thailand and the United States have demonstrated that online MSM peer outreach can result in increased use of HIV testing, particularly among those that are new to HIV services and not already in contact with HIV outreach interventions 17, 18, 19.

In 2015, shortly after adopting the global 90‐90‐90 goals, the Vietnam MOH embraced new strategies to increase HIV testing uptake and linkages to treatment among MSM and other key populations (KP). These new HIV testing modalities focused on community‐based organization (CBO) delivered lay provider testing and HIVST promoted through online and face‐to‐face peer outreach. The interventions were implemented as part of the USAID/PATH Healthy Markets project funded by the US President's Emergency Plan for AIDS Relief (PEPFAR) through the US Agency for International Development (USAID). This paper describes the methods, results, and implications of these new HIV testing approaches reaching MSM in two major cities of Vietnam.

## 2 Methods

### 2.1 Intervention description

In November 2015, staff from seven MSM‐led CBOs in HCMC and five in Hanoi were trained to offer HIV lay provider testing, and began offering services to MSM from December 2015. The three‐day training course equipped MSM lay testers to use and interpret a rapid blood‐based professional use test (Alere Determine™ HIV‐1/2), and in steps to follow if a test was HIV‐reactive or invalid. Lay testers subsequently received supportive supervision and were assisted to master testing offered in a community setting (at CBO offices or community‐based events).

Staff from the same 12 MSM CBOs were trained in May 2016 to offer HIVST information and support to clients, where needed, and what steps to take if the self‐test was HIV‐reactive. CBOs offered clients a choice of either a blood‐based (Alere Determine™ HIV‐1/2) or oral fluid (OraQuick^®^ Rapid HIV‐1/2 Antibody) assay. Both tests were made available in the CBO office and clients could opt to test privately without guidance or communication with staff, or could request help in specific steps of self‐testing. Vietnamese language test inserts, pamphlets and tutorial videos were developed to support self‐test performance. MSM with an HIV‐reactive result from peer testing or self‐testing (for those that disclosed their results) were accompanied by CBO staff for confirmatory testing at the closest district health centre, and those that were HIV diagnosed were helped with ART enrolment.

An intensive branded promotional campaign accompanied these new services, utilizing Facebook, Grindr, and MTV. A Facebook page, Rainbow Village, with 230,000 followers as of December 2017, is an open space for MSM to connect and exchange information on HIV, sexuality and wellbeing. The majority of fans are young (88% between 13 and 24) MSM and live in urban areas 20. In March 2016, eight MSM social network influencers were identified and trained to communicate directly with Rainbow Village followers and provide HIV risk screenings and referrals for HIV lay or self‐testing. To further increase ease of HIV testing uptake, a service booking application, I Reserve, was launched in February 2017. With I Reserve, MSM can quickly select where and when to test; and receive a test voucher with a unique identifier. The CBO receives an automatic booking notification and can then track test results and ART enrolment.

### 2.2 Study design

We conducted a descriptive analysis of MSM first‐time HIV testers seeking HIV lay or self‐testing as part of a larger implementation science study in Vietnam that assessed the acceptability, feasibility and effectiveness of linkages to care of HIV lay and self‐testing among KP (MSM, PWID, FSW) and their sex partners in four provinces (HCMC, Hanoi, Dien Bien and Nghe An) from December 2015 to September 2017. The larger study involved: observation and validation of lay‐provider testing performance; validation of HIV self‐test result interpretation; one cross‐sectional survey among KP that used HIV lay‐testing (n = 918) and one for KP that self‐tested (n = 936); analysis of linkage to care (test‐diagnose‐enroll) HIV cascade monitoring data; and focus group discussions with health managers, lay providers and testers. Preliminary results from the overall study including lay‐provider and self‐tester performance, HIV testing uptake and preferences, and aggregate linkage to care results have been published elsewhere 21, 22.

The primary outcome of interest for our analysis was the proportion of MSM first‐time HIV testers opting for HIV lay or self‐testing and factors that were associated with first‐time testing. We also assessed MSM satisfaction with HIV lay or self‐testing, and testing location and provider preferences and how they had heard of lay‐testing. Finally, we calculated linkage to care: the HIV positivity rate of MSM seeking HIV lay or self‐testing, and the number and proportion of those diagnosed that were enrolled in ART services. We also assessed linkage to care specifically for MSM that HIV lay or self‐tested through a social media‐based referral or booking (Table 1).

**Table 1:** Outcome measure definitions and data source

**1.** First‐time testers	MSM that report HIV testing for the first time ever	Cross‐sectional survey
**2.** HIV testing preferences	MSM preferences related to HIV testing type, location and provider	Cross‐sectional survey
**3.** Linkage to care	MSM who test HIV‐reactive that are diagnosed and enrolled in ART services	Quarterly service monitoring data

### 2.3 Participants, recruitment and data collection

Men aged 18 and above, who had sex with a man in the last 12 months, were HIV‐negative or status‐unknown, and opted for HIV lay or self‐testing were eligible for the HIV lay‐tester or self‐tester cross‐sectional study.

MSM cross‐sectional survey participants were selected during two rounds of observation of MSM lay‐provider testing performance. Each MSM tester was observed at two separate time points one week and one month after completing training. MSM were informed of the study through CBO and social media promotion. Those that were interested in coming for testing during the two observation sessions were informed about the study, its purpose and that it would involve an observer who would evaluate the performance of their lay tester and a short survey on their testing experience. After being briefed, MSM that opted to participate in the study and presented at the CBO for testing completed the informed consent process where the study's purpose, process, and potential risks and benefits were discussed. Trained research staff conducted the interviews using computer‐assisted personal interviewing (CAPI).

For the HIVST survey, a “take all” method was applied where every client who met the above mentioned criteria, accepted HIV self‐testing and opted to participate in the study were invited to join and then completed a paper‐based self‐administered survey. The HIV lay and self‐testing cross sectional survey included socio‐demographical information, HIV risk behaviours, HIV testing history, satisfaction with the testing approach; willingness to pay; and preferences related to type of HIV test, HIV tester and location of HIV testing.

The study was approved by the Institute of Social and Medical Studies IRB in Hanoi, Vietnam. PATH's research determination committee also reviewed the protocol and determined it as non‐research. Oral informed consent was obtained from all participants.

### 2.4 Analysis

The Kobo Toolbox application that hosts Open Data Kit programme (ODK) was used for the HIV lay testing CAPI survey data entry. Data were analysed using SPSS Version 22.0. The paper‐based HIVST surveys were entered using EpiData Version 3.1 and then converted to SPSS for analysis.

The main outcome of interest was the proportion of MSM first‐time HIV testers opting for HIV lay or self‐testing and factors that were associated with first‐time testing. Univariable logistic regressions were conducted to identify socio‐behavioural factors independently associated with first‐time HIV testers, and analysed separately for MSM selecting HIV lay provider testing and self‐testing. Variables found to be statistically associated with a *p*‐value of <0.05 were included in the multivariable logistic regression model. The final model was generated using backward elimination of variables with a *p*‐value of <0.05. Socio‐behavioural characteristics and HIV testing satisfaction of MSM opting for HIV lay or self‐testing were explored and compared using the Wald Test.

Data to determine linkage to care were drawn from quarterly PEPFAR direct service delivery monitoring reports and included the period of December 2015 to September 2017 for lay testing, and May 2016 to September 2017 for self‐testing. We used aggregated individual tester data to assess the number and proportion of HIV‐reactive cases among those tested, the number and proportion of those that were newly diagnosed, and the number and proportion of those diagnosed that were enrolled in ART services. As part of the linkage to care cascade analysis, we explored direct referrals from MSM online influencers and MSM who booked an HIV test using the I Reserve app. The linkage to care cascade data were analysed and visualized applying the 2015 LINKAGES guide 23.

## 3 Results

### 3.1 Enrolment

A total of 548 MSM accessing HIV lay provider testing and 803 MSM seeking HIVST opted to participate in the study. Survey completion rate was high among respondents, although in the HIVST survey there were a significant number of missing responses (343/803, 42.7% missing) to the income level question. Chi‐square analysis was conducted between first time and non‐first time testers, and respondents with and without income data and no associations were found (*p* = 0.998).

### 3.2 HIV testing uptake and frequency

Among MSM that sought HIV lay testing and HIVST, 57.9% (n = 320) and 51.3% (n = 412) were first‐time testers respectively (Table 2). For MSM that had reported ever HIV testing, the majority had tested in the previous 12 months (n = 151, 64.8% and n = 303, 77.5% respectively). Median age of MSM seeking lay and HIVST were 23 and 24 respectively.

**Table 2:** Number and proportion of new and infrequent MSM HIV lay and self‐testers

Characteristics	MSM	
	Self‐testing (n = 803)	Lay‐testing (n = 548)
Ever been HIV tested		
No, this is first time	51.3%	57.9%
Yes	48.7%	42.1%
HIV tested in past 12 month	(n = 391)	(n = 233)
No	22.5%	35.2%
Yes	77.5%	64.8%

The top three sources of information where MSM had first heard of lay testing were: peer outreach workers (70.6%), friends (62.5%) and Facebook (55.1%).

### 3.3 HIV testing satisfaction and preferences

Nearly all MSM reported being satisfied with HIVST (94.8%; n = 761/803) and lay provider testing (98.2%; n = 538/548). Among MSM that sought HIV lay provider testing, 85.4% (n = 468/548) said they would opt for this form of testing in the future, while 11.5% (n = 63/548) had no preference in type of future HIV testing. For MSM that chose HIVST, 57.2% (n = 459/803) stated they would prefer CBO staff assisted self‐testing, 21% (n = 169/803) reported wanting to self‐test alone in the CBO office, and 16.6% (n = 133/803) said that their preference was to self‐test at home.

### 3.4 Factors associated with being a first‐time HIV tester opting for HIV lay provider or self‐testing

In the univariable regression for MSM selecting HIV lay testing, being a new HIV tester was associated with younger age, no sex exchanged for money, and lower income (*p* < 0.05). These associations remained statistically significant in the multivariable analysis (Table 3).

**Table 3:** Factors associated with being a first‐time HIV tester and opting for lay provider testing

Socio‐demographics	Total (n = 548)	First‐time testing (n = 316)	Ever tested (n = 232)	Univariable logistic regression		Multivariable logistic regression	
				OR (95% CI)	*p*‐value	aOR (95% CI)	*p*‐value
Age in years				1.04 (1.008 to 1.06)	**0.012**	1.03 (1.003 to 1.07)	**0.029**
Education							
High school and below	35%	108 34.2%	84 36.2%	0.91 (0.64 to 1.3)	0.623	1.05 (0.69 to 1.6)	0.8
To College and university	65%	208 65.8%	148 63.8%	1		1	
Sex exchange for money							
No	86.7%	285 90.2%	190 81.9%	2.1 (1.27 to 2.47)	**0.004**	2.3 (1.35 to 2.9)	**0.002**
Yes	13.1%	30 9.5%	42 18.1%	1		1	
Ever injected drugs							
No	95.6%	301 95.3%	223 96.1%	1		1	
Yes	4.0%	14 4.4%	8 3.4%	1.29 (0.53 to 3.14)	0.566	1.86 (0.71 to 4.87)	0.2
Income							
Below mean	55.3%	189 59.8%	114 49.1%	1.7 (1.2 to 2.4)	**0.003**	1.6 (1.1 to 2.3)	**0.013**
Above mean	38.5%	104 32.9%	107 46.1%	1		1	
Residence							
Hanoi	34.5%	110 34.8%	79 34.1%	1		1	
Ho Chi Minh City	65.5%	206 65.2%	153 65.9%	0.96 (0.67 to 1.38)	0.854	1.25 (0.82 to 1.9)	0.29

Bold denotes *p*‐value <0.05.

In the final adjusted model, the odds of being a first‐time tester were higher among MSM who had never exchanged sex for money (aOR 2.3; 95% CI: 1.35 to 2.9) or who had income below the mean level (aOR 1.6; 95% CI: 1.1 to 2.3).

In the univariable regression for MSM seeking HIVST, a lower level of attained education was associated with being a new HIV tester at *p* < 0.05 (Table 4). The odds of being a new HIV self‐tester and having attained a lower level of education remained high in the multivariable logistic regression analysis (aOR 1.8; 95% CI: 1.3 to 2.5).

**Table 4:** Factors associated with being a first‐time HIV tester and opting for self‐testing

Socio‐demographics	Total (n = 803)	First‐time testing (n = 412)	Ever tested (n = 391)	Univariable logistic regression		Multivariable logistic regression	
				OR (95% CI)	*p*‐value	aOR (95% CI)	*p*‐value
Age in years				0.99 (0.996 to 1.03)	0.87	0.98 (0.95 to 1.02)	0.45
Education							
High school and below	41.1%	201 (48.8%)	129 (33.0%)	1.9 (1.4 to 2.3)	**0.000**	1.8 (1.3 to 2.5)	**0.001**
College and university	58.9%	211 (51.2%)	262 (67.0%)	1		1	
Sex exchanged for money							
No	70.6%	277 (67.2%)	290 (74.2%)	0.72 (0.5 to 1.0)	0.081	0.83 (0.57 to 1.2)	0.34
Yes	19.3%	88 (21.4%)	67 (17.1%)	1		1	
No response	10.1%	47 (11.4%)	34 (8.7%)	1.05 (0.61 to 1.8)	0.85	1.0 (0.57 to 1.8)	0.9
Ever injected drugs							
No	94.8%	383 (93.0%)	378 (96.7%)	1		1	
Yes	1.6%	9 (2.2%)	4 (1.0%)	2.22 (0.67 to 7.27)	0.187	2.2 (0.69 to 7.6)	0.17
No response	3.6%)	20 (4.9%)	9 (2.3%)				
Income							
Below mean	41.0%	167 40.5%	162 41.4%	0.92 (0.61 to 1.3)	0.711	0.78 (0.51 to 1.2)	0.27
Above mean	16.3%	69 16.7%	62 15.9%	1		1	
No response	42.7%	176 42.7%	167 42.7%	0.94 (0.63 to 1.4)	0.79	0.84 (0.55 to 1.2)	0.42
Residence							
Hanoi	35.7%	139 33.7%	148 37.9%	1		1	
Ho Chi Minh City	64.3%	273 66.3%	243 62.1%	1.19 (0.89 to 1.59)	0.224	0.99 (0.72 to 1.3)	0.9

Bold denotes *p*‐value <0.05.

### 3.5 Linkage to care cascade results

From December 2015 to September 2017, 23,180 MSM received HIV lay testing. There were 1582 HIV‐reactive cases (6.8%), of which 1386 were HIV‐diagnosed (87.6%) and 1290 (93.1%) were enrolled in ART (Figure S1).

There were an additional 4220 MSM that opted for HIVST from May 2016 to September 2017. Among those, 297 (7%) tested HIV‐reactive and 269 (90.6%) were confirmed HIV‐positive. Of those diagnosed, 243 (90.3%) were registered for treatment (Figure S2).

**Figure 1:**
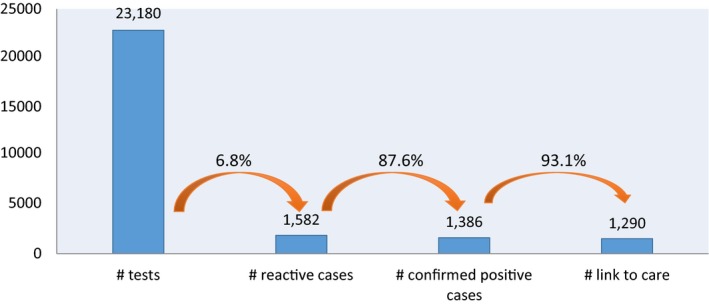
HIV lay‐testing cascade among men who have sex with men, January 2016 to September 2017

**Figure 2:**
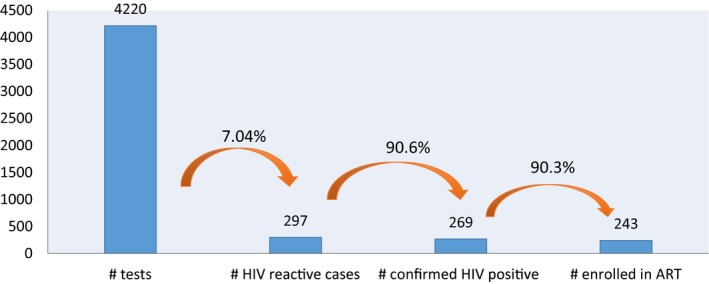
HIV self‐testing cascade among men who have sex with men, May 2016 to September 2017

As part of these linkage to care cascades, 2067 MSM were counselled and screened for HIV risk by social media influencers, 1429 (69.1%) were referred for an HIV test based on their HIV risk profile, 1419 tested (99.3%) and 10.9% (n = 155) were HIV diagnosed. A total of 387 MSM used the newly launched I Reserve app, 367 were HIV tested (94.8%), and 11.1% (n = 43) were HIV diagnosed. All 198 of those diagnosed were enrolled in ART services (Figures 1 and 2).

## 4 Discussion

Despite Vietnam MOH recommendations that MSM and other key populations test for HIV at minimum annually, uptake has been consistently poor. Our results demonstrate substantial use of and preference for HIV lay‐provider and self‐testing among MSM, particularly in young, and new or infrequent HIV testers. In a 22‐month period, 12 MSM‐led CBOs in two cities successfully tested 27,000 peers, identified 1655 HIV cases and linked the majority (>90%) of them into treatment. Peer‐delivered HIV testing and self‐testing also resulted in higher detection of new HIV cases (6.8% to 11%) compared to the national rate among KP testing in conventional governmental facilities (estimated at 1.6% in 2016) in Vietnam 24. This was the first time that MSM‐led CBOs were allowed to offer such services to peers, and play an active role in the 90‐90‐90 cascade in Vietnam.

The authors are not aware of any other study that has described such a high volume of peer delivered HIV testing uptake with MSM. One study from Thailand describes a similar model to ours, where MSM were trained to offer HIV testing through five CBOs in four localities. While the scale was much smaller—in 16 months, the CBOs tested 1680 individuals—the HIV diagnosis and ART enrolment rates were similarly high 25, 26.

We found that HIV lay and self‐testing were very effective at reaching new MSM HIV testers (more than 50% for both testing modalities). While we were not able to identify studies that measured impact of MSM‐delivered HIV testing on new tester uptake, a pooled analysis of 12 studies that measured the impact of HIVST on new HIV testing, reported that 18.7% of MSM were first‐time testers (9.9% in higher‐income countries; 32.9% in lower‐income countries) 27. The proportion of new HIV self‐testers in our study was more than twice the pooled average, and nearly double that of lower income countries reported in this meta‐analysis. Combined, new MSM testers that opted for HIV lay and self‐testing were more likely to be young, have a lower income, not have exchanged sex for money, and have attached lower levels of education. Studies in higher and lower‐income countries have identified younger age and lower education attainment with never HIV testing 28, 29, 30. This suggests that HIV lay and self‐testing are reaching at‐risk MSM in a way that traditional HIV testing services are not equipped to.

In our study, we found that young MSM were more likely to select HIV lay provider testing or self‐testing; the majority were under 30 years of age. This may be in part due to the blend of online and traditional promotion of the services. Although the number of MSM reached online and linked to testing were minimal (6.6% of total), more than half of lay‐testers stated that they were informed of the service through Facebook. Importantly, online outreach and use of I Reserve HIV service booking app resulted in a near doubling of HIV‐positivity in MSM testers. The HIV positivity yield derived from these approaches was almost the same, but I Reserve may be easier to scale given it is an individually driven tool, and no or minimal peer interaction is required. While our results suggest online reach as an important strategy for identifying newer, higher‐risk or otherwise “hidden” populations, moving forward, experimental studies measuring the effectiveness of social media interventions on MSM uptake and frequency of HIV testing in Vietnam should be conducted.

MSM testers reported a high degree of satisfaction with these new models of CBO‐delivered testing, and intent to re‐test and refer peers. Given the range of MSM HIVST‐delivery preference we found in our study, from CBO‐assisted, to unassisted home testing, it will be important to continue to offer a range of options that maximize acceptability and uptake. While the majority of respondents stated preference for CBO‐assisted HIVST, it is possible this will shift to greater interest in unassisted HIVST as these modals become more widely available and as MSM in Vietnam become more familiar with HIV self‐tests and how they work.

There are several limitations associated with the data presented in this paper. First, as a descriptive study, we were not able to directly compare MSM using HIV lay and self‐testing to facility‐based testing, and assess differences in the proportion of first‐time HIV testers, HIV positivity yield and linkage to care between the two. Second, our study was not based on a probability sample, and was not necessarily representative of MSM in HCMC and Hanoi. Third, in a bid to keep the self‐administered HIVST survey short and encourage data completion we limited variables and did not include questions on whether it was social media content, face‐to‐face interactions or both that prompted HIVST uptake.

Despite these limitations, the large number of MSM that were new to HIV testing that actively sought out HIV lay and self‐testing from MSM‐led CBOs is encouraging. These findings suggest that HIV lay and self‐testing should be scaled to other geographies in Vietnam, while continuing to evaluate and refine newer approaches to reach high risk MSM that are still reluctant to seek HIV testing, such as older MSM. This could include assessing preferences for and uptake of unassisted HIVST through online, pharmacy and partner distribution models; and measuring the acceptability and impact of partner notification and index testing among the sex and drug using partners of newly HIV‐diagnosed MSM. Although not measured in this study, linkages to HIV prevention services, particularly pre‐exposure prophylaxis (PrEP), is a critical component of HIV testing services for those at high risk testing HIV‐negative. With it now available in Vietnam 31, strength of linkages between HIV lay and self‐testing and PrEP should be evaluated.

The results from our study have important public health implications and suggest that if Vietnam were to scale MSM peer delivered HIV testing and self‐testing, it could accelerate progress towards epidemic control by more effectively identifying new HIV infections, more rapidly linking them to treatment, and critically, reducing onward transmission. Given the demonstrated ability of MSM CBOs in Vietnam to offer quality, convenient and confidential HIV testing services, we feel that this approach could be applied in other countries with similar contexts where MSM HIV testing‐uptake is static and/or low.

## 5 Conclusions

HIV lay provider and self‐testing, promoted through social media and traditional outreach, resulted in significant uptake among young, previously HIV testing naïve MSM in two major cities in Vietnam. These community‐delivered HIV testing approaches should be included in national HIV testing guidelines in Vietnam and substantially scaled to increase MSM use of HIV testing, and early access to treatment and PrEP. Without MSM‐led and acceptable HIV testing approaches such as these, it is unlikely that Vietnam will achieve 90‐90‐90 by 2020 and epidemic control by 2030.

## Competing interests

The authors have no competing interests to declare.

## Authors’ contributions

KEG, BVU, HTTP and MHT were study co‐principal investigators. KEG, BVU and MHT drafted the manuscript with input from the co‐authors. SHV and TMN provided advice and guidance on the intervention design. HVN, AHD, AB, TTT, TNNN and LTXH provided intervention oversight, training and quality assurance. MHT, TML and TTD provided data collection oversight. MHT, LHD and GTTH conducted data analysis. All authors have read and approved the final manuscript.

## Funding

USAID funded the HIV testing interventions and study.

## Supporting information


**Figure S1.** Age distribution among men who have sex with men who opted for lay provider testing.

**Figure S2.** Age distribution among men who have sex with men who opted for self‐testing.
