# Supplementary figures and images for: From conventional to disruptive: upturning the HIV testing status quo among men who have sex with men in Vietnam
Source: J Int AIDS Soc. 2018 Jul 22;21(Suppl Suppl 5):e25127. doi: 10.1002/jia2.25127 (PMC6055123; doi:10.1002/jia2.25127)

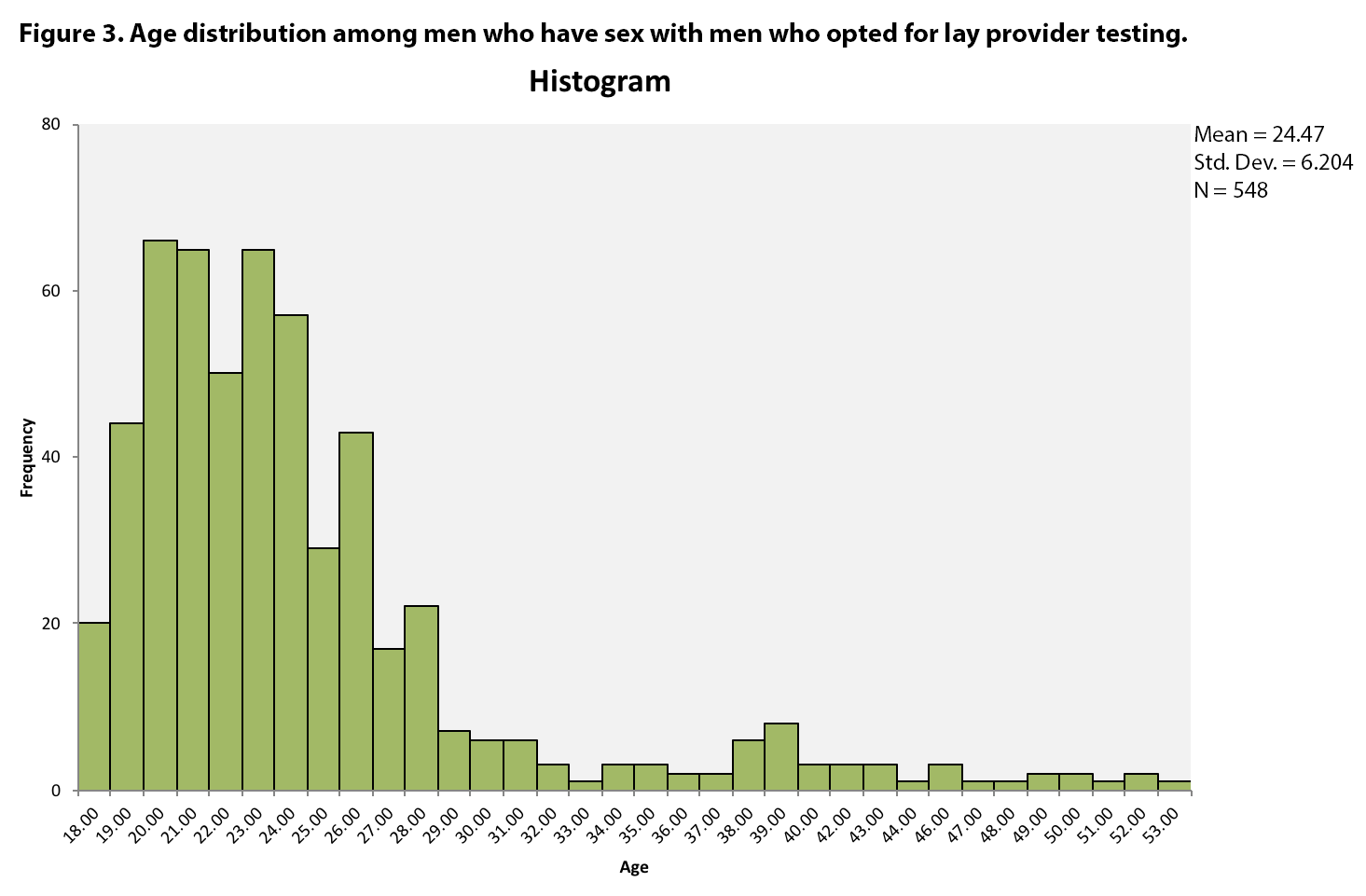

Supplement: Supplementary file 1 — Figure S1. Age distribution among men who have sex with men who opted for lay provider testing. [file JIA2-21-e25127-s001.png]

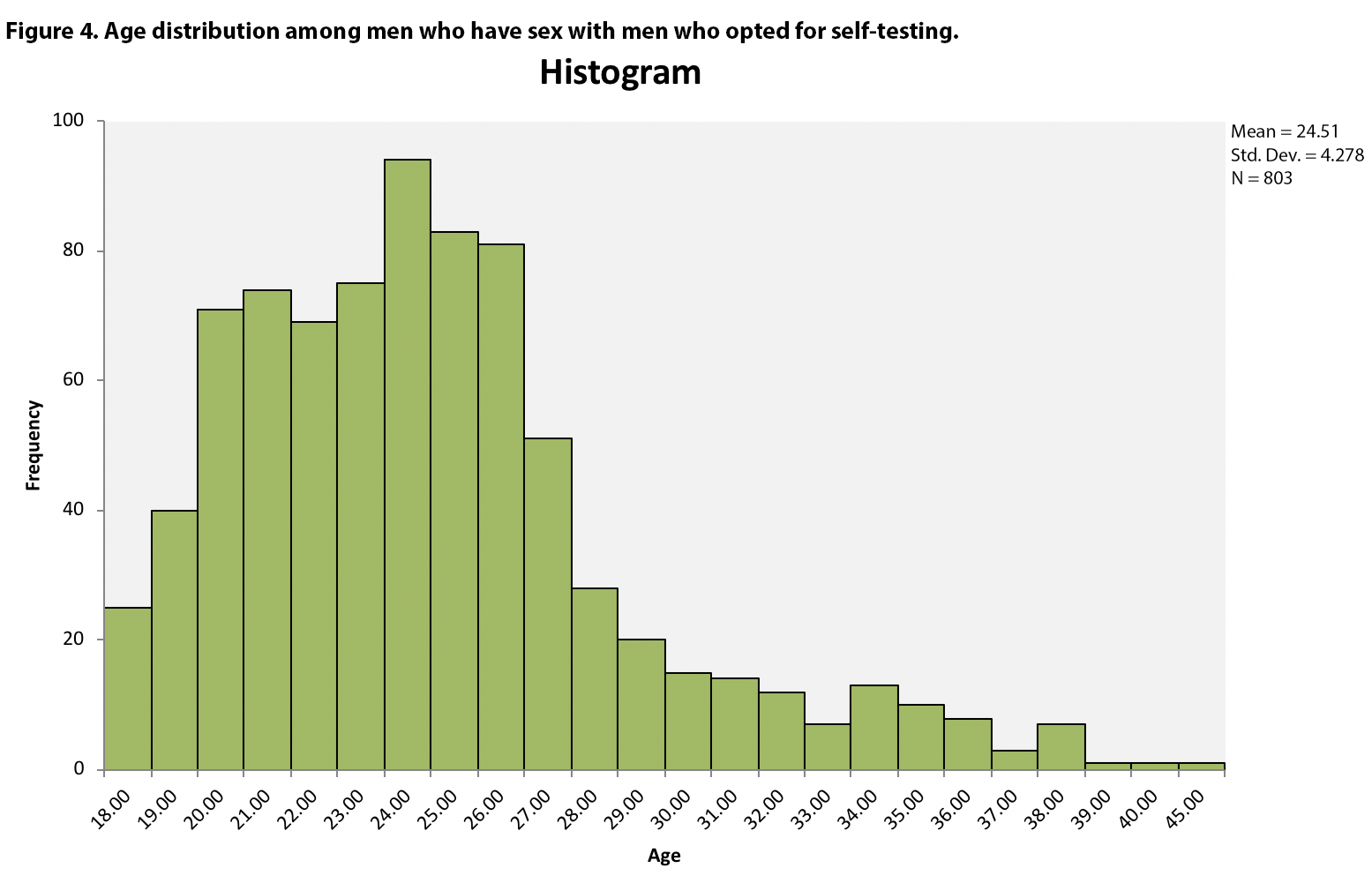

Supplement: Supplementary file 2 — Figure S2. Age distribution among men who have sex with men who opted for self‐testing. [file JIA2-21-e25127-s002.png]
